# Genome-wide analysis and expression profiling of the *PIN* auxin transporter gene family in soybean (*Glycine max*)

**DOI:** 10.1186/s12864-015-2149-1

**Published:** 2015-11-16

**Authors:** Yongqin Wang, Chenglin Chai, Babu Valliyodan, Christine Maupin, Brad Annen, Henry T. Nguyen

**Affiliations:** Division of Plant Sciences and National Center for Soybean Biotechnology, University of Missouri, Columbia, MO 65211 USA

**Keywords:** Soybean, *Glycine max*, *PIN*, Auxin efflux carriers, Polar auxin transport, ABA, Abiotic stresses

## Abstract

**Background:**

The plant phytohormone auxin controls many aspects of plant growth and development, which largely depends on its uneven distribution in plant tissues. Transmembrane proteins of the PIN family are auxin efflux facilitators. They play a key role in polar auxin transport and are associated with auxin asymmetrical distribution in plants. *PIN* genes have been characterized in several plant species, while comprehensive analysis of this gene family in soybean has not been reported yet.

**Results:**

In this study, twenty-three members of the *PIN* gene family were identified in the soybean genome through homology searches. Analysis of chromosome distribution and phylogenetic relationships of the soybean *PIN* genes indicated nine pairs of duplicated genes and a legume specific subfamily. Organ/tissue expression patterns and promoter activity assays of the soybean *PIN*s suggested redundant functions for most duplicated genes and complementary and tissue-specific functions during development for non-duplicated genes. The soybean *PIN* genes were differentially regulated by various abiotic stresses and phytohormone stimuli, implying crosstalk between auxin and abiotic stress signaling pathways. This was further supported by the altered auxin distribution under these conditions as revealed by *DR5::GUS* transgenic soybean hairy root. Our data indicates that *GmPIN9*, a legume-specific *PIN* gene, which was responsive to several abiotic stresses, might play a role in auxin re-distribution in soybean root under abiotic stress conditions.

**Conclusions:**

This study provided the first comprehensive analysis of the soybean *PIN* gene family. Information on phylogenetic relationships, gene structure, protein profiles and expression profiles of the soybean *PIN* genes in different tissues and under various abiotic stress treatments helps to identity candidates with potential roles in specific developmental processes and/or environmental stress conditions. Our study advances our understanding of plant responses to abiotic stresses and serves as a basis for uncovering the biological role of *PIN* genes in soybean development and adaption to adverse environments.

**Electronic supplementary material:**

The online version of this article (doi:10.1186/s12864-015-2149-1) contains supplementary material, which is available to authorized users.

## Background

Plant phytohormones are small signaling molecules that are synthesized within plant and control many aspects of plant growth and development, as well as plant responses to environmental cues. Auxin is the most studied and the most important plant hormone. It plays crucial roles in apical meristem maintenance, axillary meristem formation, growth, as well as phototropism, gravitropism and hydrotropism. Executing the multiple roles of auxin is largely dependent on its uneven distribution in plant, which is achieved through an active process called polar auxin transport mediated by plasma membrane auxin transporter proteins [1, 2]. Three major gene families of auxin transporters have been found in plants. Of them the plant specific PIN-FORMED (PIN) auxin efflux facilitators are key players in this process, which work together with the AUXIN1 (AUX1)/LIKE AUX1 (LAX) influx carriers and the phosphoglycoprotein (PGP/MDR⁄ABCB) efflux/influx transporters [3, 4]. The asymmetric subcellular localization of PIN proteins determines the directionality of intercellular auxin flow and the differential distribution of auxin within plant tissue, thereby controlling various plant developmental processes [3, 5, 6].

Our current knowledge about PIN-dependent polar auxin transport in plant mostly comes from the extensive investigation of the PIN gene family in Arabidopsis (*Arabidopsis thaliana*), which includes 8 members [7]. Five of the Arabidopsis PINs (PIN1-4 and PIN7) are located in the plasma membrane and they play a prominent role in the directional, cell-to-cell auxin transport [6]. Their spatiotemporal expression patterns and the auxin-dependent cross regulation of their expressions make them functionally redundant and complementary in a variety of plant developmental processes, including embryogenesis, organogenesis, tissue differentiation and tropism [8]. On the contrary, the endoplasmic reticulum (ER)-localized PIN5, 6, and 8 are not directly involved in the cell-to-cell auxin transport, but play a role in intracellular regulation of auxin homeostasis by working together with members of the PIN-LIKE auxin efflux carriers [9–13]. Besides their obvious role in many developmental processes, results from Arabidopsis show that PIN proteins are also involved in the crosstalk of auxin, ethylene, cytokinin and strigolactone in root development, where PIN proteins serve as a playground for the integration of hormone signaling through regulation of intracellular PIN protein trafficking and then subcellular polar localization [14]. Recently, the *PIN* gene family has been characterized from rice, sorghum, maize, and potato [15–19]. Transcriptional profiling analyses in rice, sorghum and maize suggested that some *PIN* genes from these plant species might mediate the crosstalk between auxin, other hormones and abiotic stresses [15–17].

Soybean is one of the most widely grown crops in the world. It is the most important source of vegetable protein and oil for humans, the most preferable protein source for farm animals, and currently the major feedstock for biodiesel production. With the rapid growth of global population and environmental degradation, improving soybean yield is a crucial task to meet the human demand for food and energy. Considering the importance of *PIN* genes in plant growth regulation and in plant response to abiotic stress environments, we carried out genome-wide comprehensive analysis of the soybean *PIN* auxin efflux transporter gene family. Their tissue expression patterns and expression profiling under hormonal treatments such as auxin and abscisic acid (ABA), and abiotic stresses including drought, salt and dehydration were analyzed. Our research identified the soybean *PIN*s associated with abiotic stress responses. Some of them might be ideal candidates for further investigation.

## Results and Discussion

### Identification and phylogenetic analysis of the soybean *GmPIN*s

Twenty-four putative *GmPIN* loci have been found through BLAST searches of the *Glycine max* reference genome (v1.1) by using *A. thaliana* PIN protein sequences, including two truncated loci, Glyma13g09026 and Glyma13g09043, which were located in the same region of the chromosome. They were treated as a single locus (Glyma13g09030) in the annotation of *Glycine max* version 1.0. We adopted the gene model of Glyma13g09030 in our following analysis because Glyma13g09026 (encoding a 126-amino acid peptide) and Glyma13g09043 (encoding a 350-amino acid peptide) show high sequence similarities with the C terminal and N terminal of Glyma14g27900, respectively. These two loci might be evolved from a single locus, and further experiments are needed to verify this gene model. Therefore, a total of 23 members of the soybean *PIN* family have been identified. Using the same approach, 16, 12 and 5 putative *PIN* members were identified from common bean (*Phaseolus vulgaris*), *Medicago truncatula* and *Lotus japonicus*, respectively*.* Besides the previous characterized *MtPIN1*-*7* [20], five new full-length sequences were identified in the *Medicago truncatula* v4 release.

**Fig. 1:**
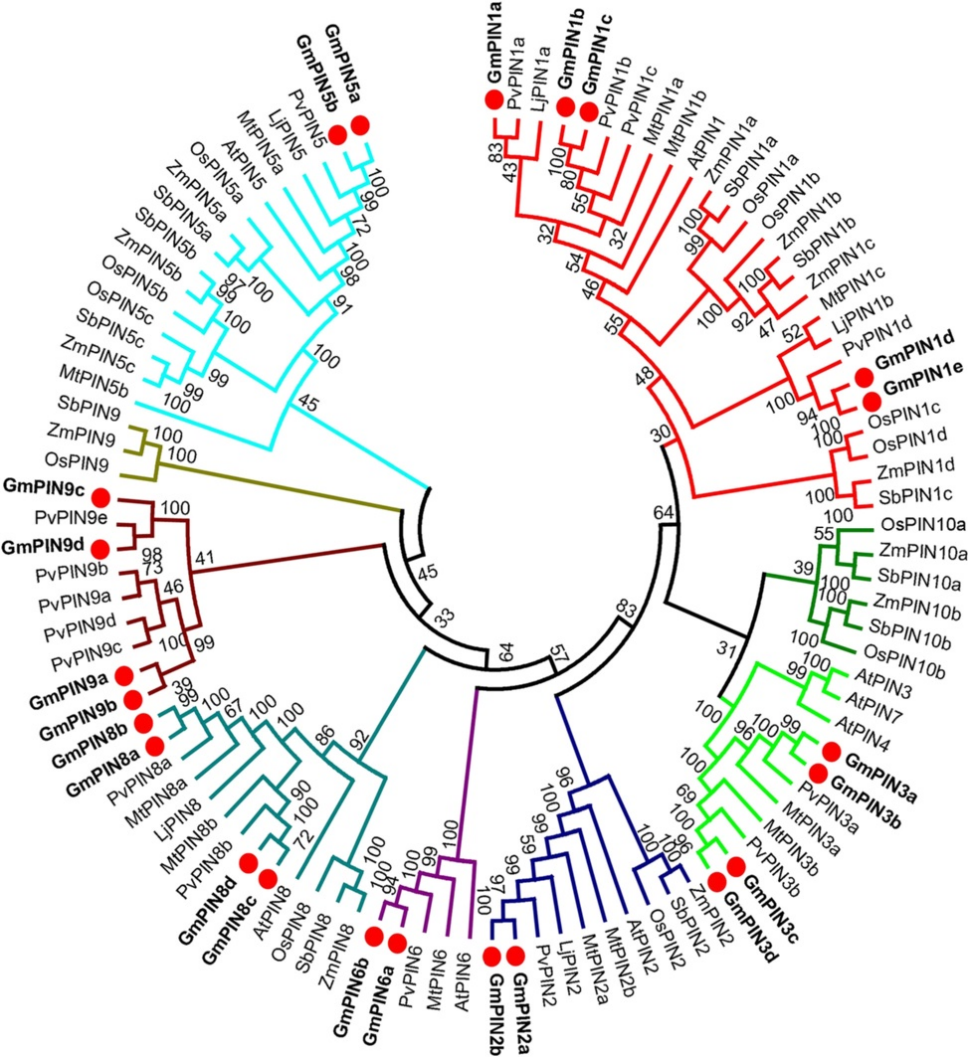
Phylogenetic relationships of the PIN auxin transporters from soybean (*Gm*), common bean (*Pv*), *Medicago truncatula* (*Mt*), *Lotus japonicus* (*Lj*), Arabidopsis (*At*), rice (*Os*), maize (*Zm*), and sorghum (*Sb*). The phylogenetic tree was constructed using Mega 5.2 [43]**.** Accession numbers of the PIN proteins are reported in Additional file 3: Table S2. The 99 PIN proteins from 8 plant species can be divided into 7 groups (subfamilies), branches of which are differently colored. The soybean genes are shown in bold font

A phylogenetic tree was built with 99 protein sequences from 8 plant species in order to investigate the phylognetic relationships among PINs from soybean, three other legumes, Arabidopsis and three grasses (Fig. 1). The protein sequences from Arabidopsis, rice, sorghum and maize were retrieved from the publications [7, 15–17]. Genes from soybean, common bean, *Lotus japonicus, Medicago truncatula* and sorghum were named according to the cluster of the PIN family from Arabidopsis, rice, and maize. The analysis revealed that these PINs could be divided into 7 groups (subfamilies): PIN1, PIN2, PIN3 (dicot-specific)/PIN10 (monocot-specific), PIN5, PIN6, PIN8, and PIN9. No members have been found in the PIN6 group from the three grasses. The PIN9 group from legumes and grasses, and the dicot-specific PIN3 and the monocot-specific PIN10, might have evolved independently, respectively. Interestingly, the specific functions of these exclusive PINs are still not clear. Within other groups, PINs from monocots and dicots are clustered separately, and the legume PINs show a very close evolutionary relationship (excluding MtPIN5b from *Medicago truncatula*).

Compared to other species, the soybean *PIN* gene family is extensively expanded, having approximately twice the number of *PIN*s in rice, maize, sorghum, and *Medicago truncatula* (Table 1). Expansion of the soybean *PIN* gene family could be due to the two whole-genome duplication events, with one occurring approximately 59 million years ago shared by soybean, common bean, and medicago and the other glycine-specific one around 13 million years ago [21–23]. We noticed that tandem duplication was another factor attributing to evolution of the common bean *PIN* gene family: a cluster with three tandem genes (*PvPIN9b*-*d*) was found within a 20 kb region on chromosome 9. However, no tandem duplication was observed in the *PIN* genes from soybean.

**Table 1:** Number of *PIN* genes in eight plant species

Species	Long PIN					Short PIN		
	PIN1	PIN2	PIN3/10	PIN6	PIN9	PIN5	PIN8	Total
Soybean	5	2	4	2	4	2	4	23
Common bean	4	1	2	1	5	1	2	16
Medicago	3	2	2	1		2	2	12
Lotus	2	1				1	1	5
Arabidopsis	1	1	3	1		1	1	8
Rice	4	1	2		1	3	1	12
Maize	4	1	2		1	3	1	12
Sorghum	3	1	2		1	3	1	11

### Chromosomal distribution, gene structure and protein profiles of *GmPIN*s

**Fig. 2:**
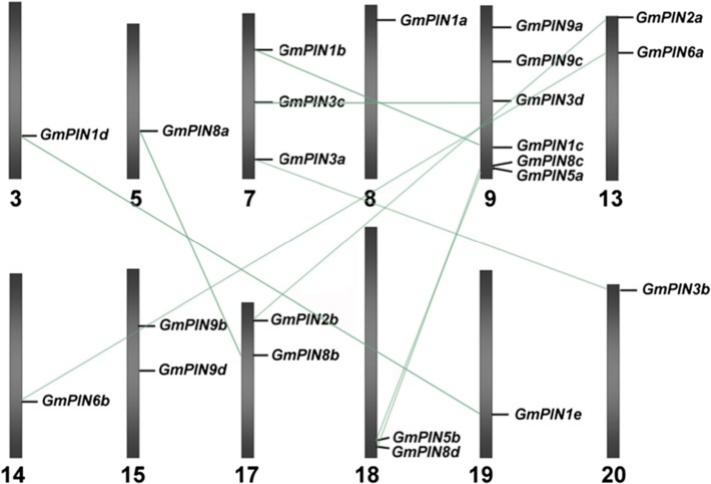
Chromosomal distributions of the identified soybean *PIN* genes. Chromosomal locations were shown from top to bottom on corresponding chromosomes according to soybean genome annotation v1.1. Duplicated gene pairs were linked by light green lines

Chromosome mapping revealed that the 23 *GmPIN*s were not evenly distributed on 12 chromosomes (Fig. 2). The gene number on each chromosome varied from one to six, with one gene on chromosomes 3, 5, 8, 14, 19 and 20 each, two genes on chromosome 13, 15, 17 and 18 each, three genes on chromosome 7, and 6 genes on chromosome 9. Duplication analysis identified 9 pairs of duplicates, which shared more than 90 % nucleotide sequence identity each and were located in duplicated blocks on 9 chromosomes. The duplicated pairs are *GmPIN1b*-*1c*, *GmPIN1d*-*1e*, *GmPIN2a*-*2b*, *GmPIN3a*-*3b*, *GmPIN3c*-*3e*, *GmPIN5a*-*5b*, *GmPIN6a*-*6b*, *GmPIN8a*-*8b*, *GmPIN8c*-*8d*, and *GmPIN9c*-*9d*.

**Fig. 3:**
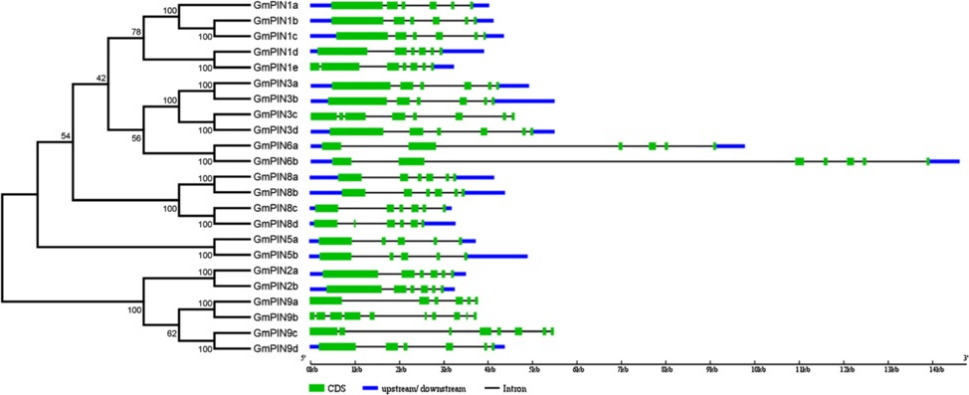
Phylogenetic relationships and gene structures of *GmPIN*s. The phylogenetic tree (left panel) was constructed using MEGA 5.2 [43], and the gene structures (right panel) were drawn using the Gene Structure Display Server [44]

The gene structure is highly conserved for most *GmPIN*s. Seventeen out of 23 contain 6 exons (Fig. 3). This conserved exon/intron organization of *PIN* genes was also found in other plant species [5, 16–19]. The exon numbers for *GmPIN1e*, *GmPIN3c*, *GmPIN6b*, *GmPIN5a*, *GmPIN5b*, and *GmPIN9b* are 7, 8, 7, 5, 5, and 10, respectively. Intron size was a major factor affecting the gene size. For example, the striking difference in gene size between the largest gene *GmPIN6b* (with a gene size of 14.6 kb) and the smallest gene *GmPIN8c* (with a gene size of 3.17 kb) was mainly due to the difference in total intron length (13.0 kb vs. 2.1 kb).

**Fig. 4:**
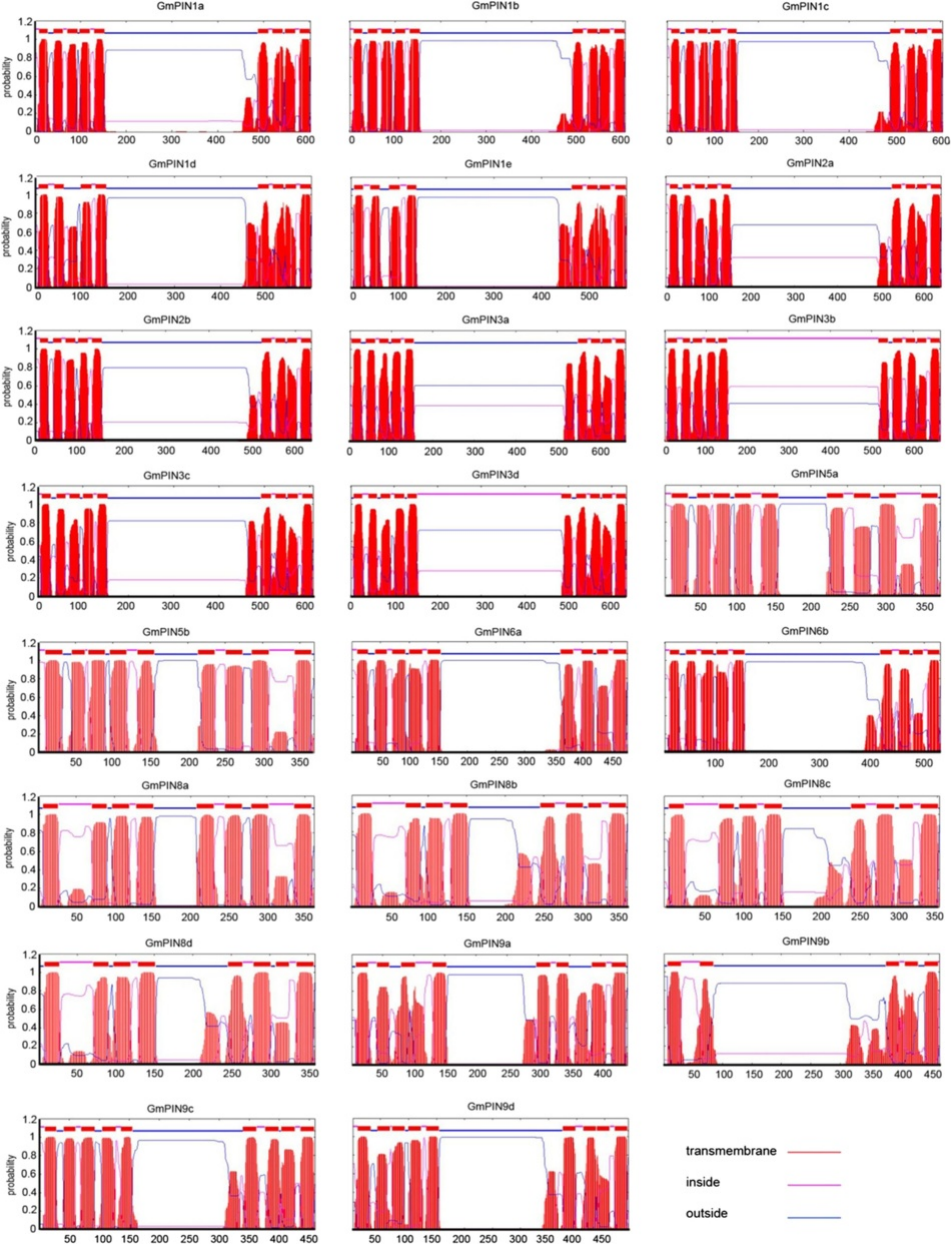
Transmembrane topology analysis of soybean GmPIN proteins. The protein transmembrane topology was predicted by using the TMHHM Server v2.0 [45]. The predicted transmembrane helices were shown as red peaks

As in Arabidopsis and other plant species [5, 7, 15–17], the soybean PINs can be grouped into long PINs and short PINs according to the predicted protein length (Table 1 and Additional file 1: Table S1). The soybean typical long PINs consist 11 members (578–666 amino acids in length), including all genes from the group PIN1, PIN2, and PIN3; while the typical short PINs comprise 6 members from group PIN5 and PIN8 (353–377 amino acids in length). A total of 6 members from PIN6 and PIN9 have a protein length (443 to 531 amino acids) between those of typical long PINs and short PINs. Similar to other plant PINs, GmPIN proteins have a highly conserved hydrophobicity profile, with two hydrophobic segments located at N- and C- termini and linked by a central hydrophilic loop (Fig. 4). All GmPIN proteins possess 8–10 transmembrane helices except for GmPIN9b, which has only 5. Multiple sequence alignment revealed that the sequences of N- and C- terminal transmembrane segments in GmPIN proteins were highly conserved, and that the central hydrophilic loop was of high heterogeneity (Additional file 2: Figure S1). The length of the central hydrophilic loop is around 300 amino acids for members of the long PINs, 50–100 amino acids for members of the short PINs, and in between for members from group PIN6 and PIN9. Investigating the subcellular localization of GmPIN proteins is very helpful for understanding their molecular function *in planta*. In soybean, most of the long PIN proteins were predicted to be localized in plasma membrane, while majority of the short ones were not (Additional file 1: Table S1). It has been reported that in Arabidopsis, the plasma membrane-localized long PINs are responsible for the cell-to-cell auxin polar transport, while ER-localized short PINs are involved in intracellular regulation of auxin homeostasis [9–12]. Their orthologous genes in soybean may have similar function.

### Tissue-specific expression profile of *GmPIN*s

**Fig. 5:**
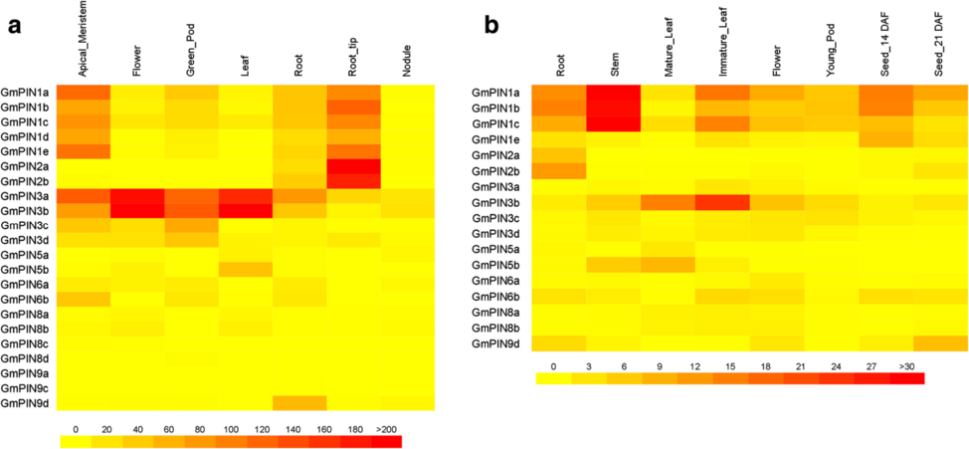
Tissue-specific expression profiles of *GmPIN* genes. (**a**) Expression profiles of *GmPIN*s in seven tissues according to RNA-Seq data [25]. The Reads/Kb/Million (RPKM) normalized values were visualized as a heat map. (**b**) Quantitative RT-PCR analysis of selected *GmPIN*s in eight soybean tissues. Relative expression levels of *GmPIN*s compared to an ubiquitin gene (Glyma20g27950.1) were multiplied by 1,000 and shown as a heat map. Data represented the means of three biological and two technical replicates

In order to investigate the tissue-specific expression pattern of *GmPIN*s, a heat-map showing the expression of 22 *GmPIN*s in seven tissues (shoot apical meristem, flower, green pod, leaf, root, root tip and nodule) was constructed (Fig. 5a) using the publically available soybean RNA-Seq data [24, 25]. No related data was found for *GmPIN9b*, and barely detectable or no expression was observed for *GmPIN8c*, *GmPIN8d*, *GmPIN9a*, and *GmPIN9c*. Gene expression was detected in at least one tissue for all other *GmPIN*s. Based on the RNA-Seq data, qRT-PCR (quantitative Reverse Transcription-Polymerase Chain Reaction) analysis was carried out to study the expression patterns of 17 *GmPIN*s in eight tissues, including root, stem, mature leaf, immature leaf, flower, pod, and seed at 14 and 21 days after flowering. Results of the qRT-PCR analyses were shown in Fig. 5b. The expression data in Fig. 5a and 5b revealed overall similar expression patterns (but at different expression levels) for duplicated *GmPIN*s or genes from the same PIN group, but very different expression profiles for *GmPIN*s from different PIN groups. Genes from the PIN1 and PIN3 groups were differentially expressed in all or most tissues tested. Transcripts for several genes from the PIN1 group were abundant in shoot apical meristem, root tip and stem, but barely detectable in nodule. *GmPIN3a* and *GmPIN3b* were highly expressed in flower and leaf, while *GmPIN3c* and *GmPIN3d* were expressed at relatively low levels in many tissues. *GmPIN2a* and *GmPIN2b* were predominantly expressed in soybean root, especially in the root tip, while low levels of *GmPIN2b* expression were also detected in flower and seed. Tissue-specific and relatively low-level gene expression was detected for genes from groups PIN5, PIN6, PIN8 and PIN9, e.g. *GmPIN5b*, *GmPIN8a* and *GmPIN8b* in leaf and flower, *GmPIN6a* and *GmPIN6b* in shoot apical meristem, green pod and root, and *GmPIN5a* in leaf and nodule. *GmPIN9d* was mainly expressed in root, seed and flower.

As shown in Fig. 5, a different combination of *GmPIN* expression was observed in each tissue types, such as members of *GmPIN1*, *GmPIN3* and *GmPIN6* in shoot apical meristem, and the homologous genes of PIN1 and PIN2 in root tip. This suggests that these *GmPIN*s work cooperatively. The dynamic temporal and spatial expression of these *GmPIN*s may be critical for the developmental process of each tissue. Though expressed in the same tissue and at the same time, the cell type-specific expression pattern, subcellular polarity and subcellular localization of those genes can be different, which has been elegantly evidenced by experiments in Arabidopsis [5, 7]. The similar expression patterns of duplicated genes or genes from the same PIN group indicate functional redundancy, while synergistic expression of *GmPIN*s from different group suggests functional complementation. Both might contribute to the flexibility and variation during soybean evolution. Functional redundancy could reduce the selection pressure on duplicated genes, which might lead to novel function, loss of function, or loss of expression (such as *GmPIN8c*, *GmPIN8d*, *GmPIN9a*, *GmPIN9b*, and *GmPIN9c*).

### Expression of *GmPIN*s in response to drought, salt and dehydration

Current study indicates that the *PIN* auxin efflux transporters were evolved as key players in a plant's adaptations to their growing environment by responding to environmental and endogenous signals at both transcriptional and post-transcriptional levels [26]. Drought is one of the prime abiotic stresses worldwide, and is also the major constraint to soybean production, accounting for 40 % of yield loss [27]. Salinity is another significant stressor causing serious yield loss in salt-affected area, occupying 20 % of irrigated land in the world [28].

**Fig. 6:**
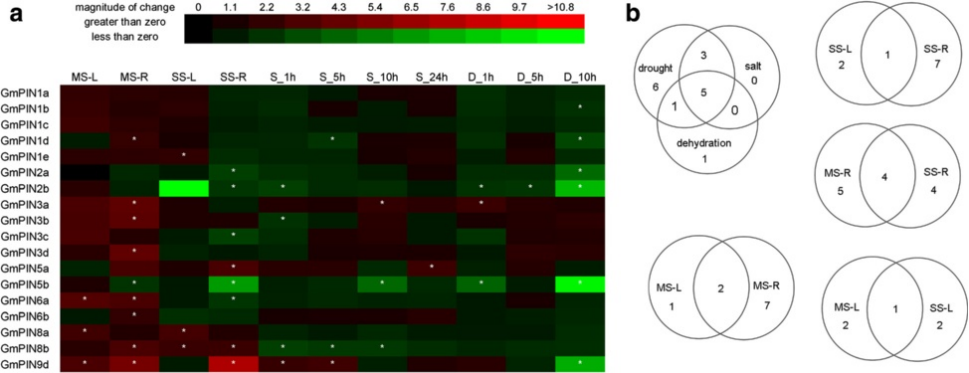
Expression profiles of *GmPIN*s under drought, salt and dehydration conditions. (**a**) Fold changes of *GmPIN* gene expression under drought, salt and dehydration stresses. Data represented the means of three biological and two technical replicates. The asterisks indicate an absolute fold change ≥ 2 and P value <0.05 by Student's t-test. (**b**) Venn diagram analysis of *GmPIN* gene expression under drought, salt and dehydration stresses. MS-L, mild drought stress shoots; MS-R, mild drought stress roots; SS-L, moderate drought stress shoots; SS-R, moderate drought stress roots; S-1 h, salt 1 hour; S-5 h, salt 5 hour; S-10 h, salt 10 hour; S-24 h, salt 24 hour; D-1 h, dehydration 1 hour; D-5 h, dehydration 5 hour; D-10 h, dehydration 10 hour

In order to explore the possible involvement of *GmPIN*s in response to water deficit conditions at the transcription level, expression profiles of 18 *GmPIN*s in soybean seedlings under drought, salt and dehydration treatments were analyzed by qRT-PCR (Fig. 6a). Significant differential expressions (fold change ≥ 2, p-value ≤ 0.05) of 16 genes were detected under one or more treatments except for *GmPIN1a* and *GmPIN1c*. Seven *GmPIN*s responded to only one stress, four genes responded to two stresses, and five genes were regulated by all three stresses (*GmPIN1d*, *GmPIN2b*, *GmPIN3a*, *GmPIN5b*, and *GmPIN9d*) (Fig. 6b). More *GmPIN*s were involved in response to drought (15 genes) than to salt stress (8 genes) and dehydration (7 genes). The number of *GmPIN*s down-regulated by salt or dehydration was larger than that of up-regulated. Of the 15 drought-responsive *GmPIN*s, five were regulated by both mild and moderate drought stresses. Interestingly, more genes were differentially regulated in root than in shoot upon either mild or moderate drought treatments (Fig. 6b). All of the differentially expressed genes in shoot tissue were induced by drought (mild or moderate), and most of those in root were up-regulated by mild drought (8 out of 9), but down-regulated by moderate drought (5 out of 8).

Our data demonstrated most *GmPIN*s were responsive to certain water deficit conditions at the transcriptional level, generally in a tissue-specific, time- and stress magnitude-sensitive mode, suggesting that soybean responds to water deficit stress through a very complex regulation network, which necessitates coordinated regulation of most *GmPIN*s. As in soybean, many *PIN* genes in sorghum and maize were found to be transcriptionally responsive to various abiotic stresses, including salt and drought [15, 17]. *PIN* genes might be commonly used for plants from different species to adapt to various abiotic stress conditions. Although a huge body of evidence has demonstrated the importance of PIN proteins in many developmental processes and in response to environmental signals such as light and gravity [1, 2, 4, 8], there is limited information on their role in abiotic stresses and the underlying molecular mechanisms. Recently, it was reported that PIN2 in Arabidopsis was required to maintain root growth under alkaline stress conditions by modulate proton [H^+^] secretion [29]. Another study indicated that the affected intracellular trafficking of PIN2 and PIN3 proteins in Arabidopsis might be responsible for the inhibited auxin polar transport under cold stress conditions [30].

### Expression of *GmPIN*s in response to ABA and auxin

**Fig. 7:**
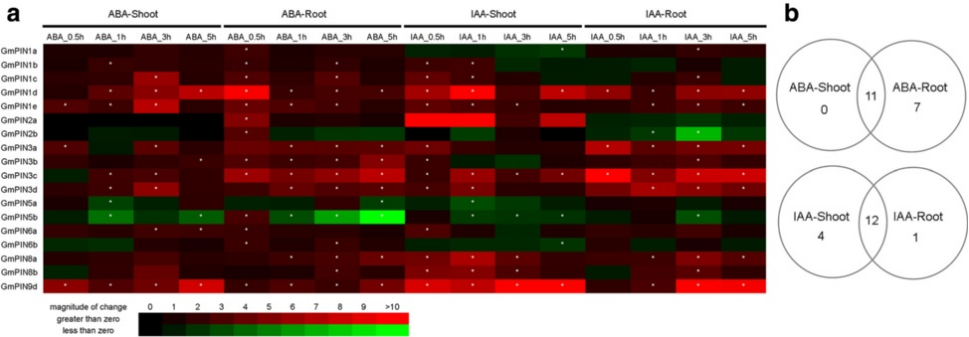
Expression patterns of *GmPIN*s under ABA and auxin treatments. (**a**) Fold changes of *GmPIN* gene expression under ABA and IAA treatments. Data represented the means of three biological and two technical replicates in qRT-PCR analysis. Asterisks indicated an absolute fold change ≥ 2 and P value <0.05 by Student's t-test. (**b**) Venn diagram analysis of *GmPIN* gene expression under ABA and IAA treatments.

The plant phytohormone ABA is pivotal for plant growth and development at its basal level and plant response to biotic and abiotic stresses at elevated levels [31, 32]. Emerging evidence indicates that auxin might play the role as mediator of environmental adaptions in plants [33, 34]. In addition, auxin feedback regulation of *PIN* gene expression at the transcriptional level is a key mechanism in auxin distribution in Arabidopsis [1, 6, 8]. Quantitative RT-PCR analysis was carried out to investigate the effect of ABA and auxin on *GmPIN* gene expression in shoot and root tissues of soybean seedlings (Fig. 7a). All of the 18 *GmPIN*s detected were differentially expressed upon ABA treatment, with 11 of them responsive in both shoot and root, and 7 in root only (Fig. 7b). Seventeen out of 18 *GmPIN*s were responsive to indole-3-acetic acid (IAA) treatment, with 12 overlapping in shoot and root. While most of the *GmPIN*s were up-regulated in shoot and/or root by the two hormones, expression of *GmPIN5a* and *GmPIN5a* was repressed by ABA and IAA, and decreased expression of *GmPIN1a*, *GmPIN2b* and *GmPIN6b* was observed in shoot or root at some time point(s) after IAA treatment.

Exposure of plants to abiotic stress conditions elicits ABA accumulation, which then triggers a series of physiological, biological and molecular changes for plants to adapt to adverse environments. Evidence from Arabidopsis and rice supported that ABA accumulation modulated auxin transport in the root tip, which was critical for maintaining root growth under water stress condition [35]. Besides ABA and auxin, many other hormones are involved in modulating plant’s response and adaption to environmental stresses [36, 37], as well as in controlling PIN gene action in many developmental processes [6, 16, 17, 26]. Regulation of *PIN*s at the transcriptional and/or posttranscriptional level, including spatial and temporal expression pattern, subcellular polar localization, intracellular trafficking and recycling, and degradation, has been employed by plants to control many growth and developmental processes [5–8, 26]. Plants may use the same or similar mechanisms to adapt to stress conditions.

### *GmPIN* promoter activity in transgenic soybean hairy root

**Fig. 8:**
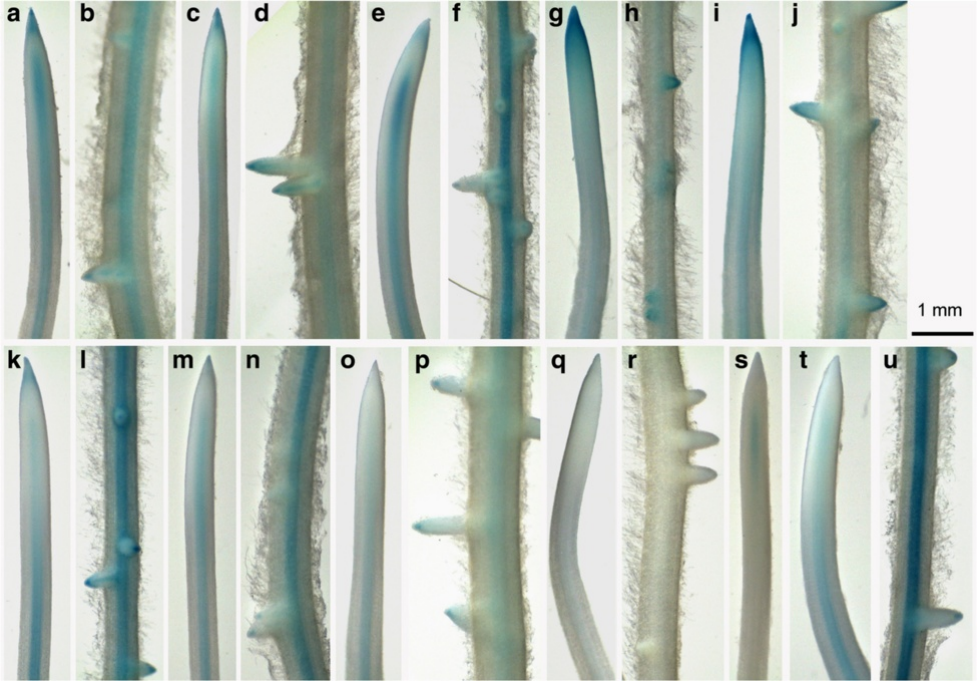
Promoter activities of *GmPIN*s in transgenic soybean hairy roots. At least 10 independent transgenic roots for each *GmPIN::GUS* construct were subjected to GUS staining. Images show representative results in root tips and lateral roots after four-hour staining for all roots except (S), which was stained overnight. (**a**) and (**b**), *GmPIN1b*; (**c**) and (**d**), *GmPIN1c*; (**e**) and (**f**), *GmPIN1e*; (**g**) and (**h**), *GmPIN2a*; (**i**) and (**j**), *GmPIN2b*; (**k**) and (**l**), *GmPIN3a*; (**m**) and (**n**), *GmPIN3b*; (**o**) and (**p**), *GmPIN6a*; (**q**), (**r**) and (**s**), *GmPIN6b*; (**t**) and (**u**), *GmPIN9d*. Scale bar, 1 mm

In order to examine gene expression pattern in soybean root, promoters of 10 *GmPIN*s were cloned and histochemical detection of promoter*::GUS* activity in transgenic soybean hairy roots was carried out (Fig. 8). Promoter activity was detected in soybean root for all of the cloned promoters in various patterns and with variable strengths, while genes from the same PIN group showed higher similarity. Strong GUS signals were observed in root cap and stele for *GmPIN1b* transgenic primary root and lateral root (Fig. 8a and 8b), *GmPIN1c* promoter showed a similar pattern of activity but weaker than *GmPIN1b* (Fig. 5c and 8d); while a much stronger *GmPIN1e::GUS* signal was found in stele than in root cap (Fig. 8e and 8f). For *GmPIN2a* and *GmPIN2b*, promoter activity was high in root cap, meristem region, lateral root tip and lateral root primordia, and weak in stele (Fig. 8g-8j). The *GmPIN3a* promoter activity was strong in lateral root cap and vascular tissue of mature root region but weak in root cap and stele of the root tip region (Fig. 8k-8l); and a similar but weak staining pattern as that of *GmPIN3a* was found in *GmPIN3b::GUS* transgenic roots (Fig. 8m-8n). For *GmPIN6a*, only weak activity was detected in stele and lateral root tips (Fig. 8o-8p); while no GUS signals were observed in *GmPIN6b::GUS* transgenic roots after staining for 4 hours (Fig. 8q-8r), but a weak signal appeared in stele of the root tip region after overnight-staining (Fig. 8s). For *GmPIN9d*, strong promoter activity was found in the vascular tissue of mature region of root, and weak activity was observed in root cap and stele of root tips (Fig. 8t-8u).

In soybean roots, the overall similar patterns in promoter activity of the duplicated genes, such as *GmPIN1b*-*1c*, *GmPIN2a*-*2b*, and *GmPIN3a*-*3b*, further suggest their function redundancy. Members from PIN1-3 groups and *GmPIN9d* may play very important roles in soybean root development due to their relatively strong expression in this tissue. Notably, the patterns of promoter activity in soybean roots for the detected *GmPIN*s were different from those of their orthologous genes in Arabidopsis and rice [8, 16]. The large gene number, various tissue-specific expression patterns and versatile regulatory modes under internal and external cues make it very complicated to explore the specific role of a certain *GmPIN* gene in soybean development. Further cellular and subcellular localization of their proteins in plant and gene-specific or a group of duplicated gene-specific knockout transgenic analysis may be helpful to unravel their functions.

### Auxin distribution and *GmPIN9d* promoter activity in soybean root in response to environmental signals

**Fig. 9:**
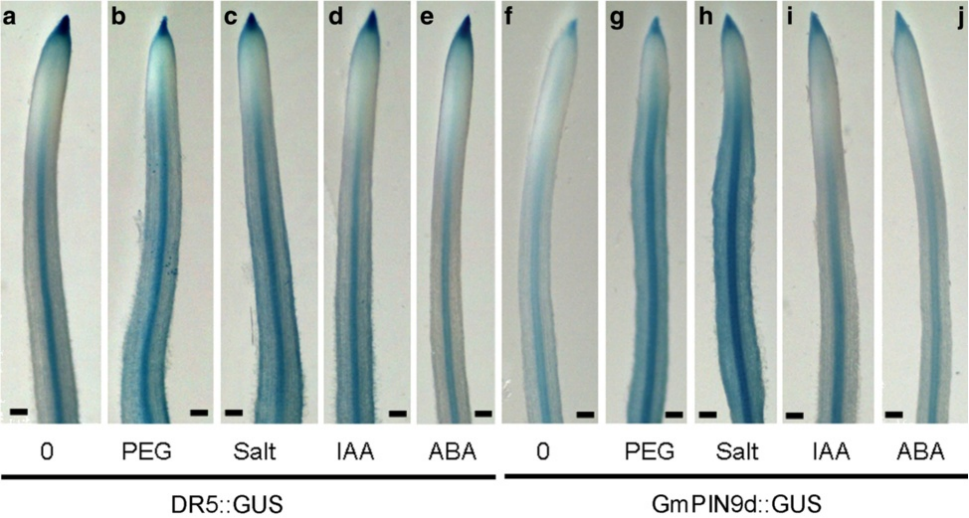
Promoter activities of *DR5* and *GmPIN9d* in transgenic soybean hairy roots under different treatments. Images show representative results of at least 5 biological replicates. Scale bar, 0.2 mm

The auxin responsive *DR5::GUS* reporter system was used to monitor the auxin distribution in soybean root in response to water stress conditions and auxin and ABA treatments (Fig. 9a-9e). Promoter activity of *GmPIN9d*, which is a legume specific PIN9 group member and responded to various treatments at the transcriptional level (Figs. 6 and 7), was also investigated under these conditions (Fig. 9f-9j). Under control condition (Fig. 9a), the maximum signal of *DR5* promoter activity exhibited in the root cap region, strong signal was also found in stele, but signal was barely detected in the region approximately 0.5 to 1.5 mm from the root tip. Upon PEG (polyethylene glycol) (Fig. 9b) and salt (Fig. 9c) treatments, staining was strongly induced in stele and other cells. GUS signal increased in the mature root region under IAA treatment (Fig. 9d), but decreased upon ABA treatment (Fig. 9e). As for *GmPIN9*, compared to the no-treatment control (Fig. 9f), promoter activity was greatly induced in root cap, stele and other cell types upon treatments with PEG and salt (Fig. 9g and 9h). It was also induced in root cap and stele by IAA (Fig. 9i), and slightly induced by ABA (Fig. 9j).

Changes in *DR5* promoter activity reflected altered auxin distribution or signaling. *DR5* and *GmPIN9* showed overall similar patterns in changes of promoter activity under PEG and salt treatments. This strongly indicates that *GmPIN9* might play a role in auxin re-distribution under these conditions, probably working together with other *GmPIN*s and auxin transporters from other gene families. Besides auxin transport, some auxin signaling components might also be involved in these responses. For example, some members of the auxin response factor transcription factor family in soybean were found transcriptionally regulated under water-deficit conditions [38].

## Conclusions

In this study, the soybean *PIN* auxin transporter gene family was comprehensively analyzed, including phylogeny, chromosomal distribution, gene structure, protein profiles, expression profiles in various tissues and under various abiotic stress conditions and hormone treatments, and promoter activity assay in transgenic soybean root. Eighteen out of the 23 members in the soybean *PIN* gene family exist as duplicated gene pairs originating from the glycine-specific whole-genome duplication event. High potential functional redundancy of duplicated genes or genes from the same PIN group was implied from high similarities of encoded amino acids, gene structure, tissue-specific expression pattern, and promoter activity in soybean root. However, the versatile differential expression modes under abiotic stress conditions indicated specific gene function at certain environmental conditions. The soybean *PIN* genes were responsive to a complex network of internal and external signals, and thereby controlled the auxin re-distribution, which finally will lead to the adjustment of growth and developmental processes to adapt to the ever-changing environments. Further in-depth functional analysis of the biological roles of *GmPIN* genes will enhance our understanding of plant response to abiotic stresses and aid in development of stress resistant crops.

## Methods

### Identification of *PIN* auxin efflux carriers from soybean and other legumes

Putative soybean and common bean *PIN* auxin efflux carriers were identified by BLAST searches against the corresponding reference genome at Phytozome (v9.1) [39] using *A. thaliana PIN* protein sequences as queries. Following this approach, putative *PIN* members were identified from the *Medicago truncatula* genome (v4) [40], and the *Lotus japonicus* genome assembly build 2.5 [41]. Protein sequences were downloaded for all identified putative *PIN*s. See Additional file 3: Table S2 for accession numbers of all sequences used in this study.

### Phylogenetic analysis and chromosomal mapping

Sequence alignments of all identified PINs from four legume species in this study and PINs with published data from Arabidopsis, rice, maize and sorghum were performed using the online software Clustal Omega [42]. Result of the sequence alignments was then used to construct the unrooted phylogenetic tree by the neighbor-joining method with a bootstrap analysis of 1000 replicates using MEGA 5.2 [43]. Chromosomal position information of *GmPIN*s was obtained from gene annotation (v1.1), and the relative localization of each *GmPIN* was drawn on their respective chromosomes from the top to the bottom.

### Gene structure and protein profile analysis

Gene exon-intron structure information of *GmPIN*s was retrieved from Phytozome v9.1, and gene structure schematic diagram was drawn by using the Gene Structure Display Server [44]. Protein transmembrane topology was predicted by using TMHHM Server v2.0 [45]. Protein length, molecular weight and isoelectric point of *GmPIN*s were analyzed by the Lasergene v7.1 software. Protein subcellular localization was predicted by WoLF PSORT [46].

### Plant growth, stress and hormonal treatments and tissue collection

The soybean cultivar, Williams 82, was used in this study for the expression profiling analysis, promoter cloning and hairy root transformation. Plants were grown under the same greenhouse conditions as reported [47]. For tissue/organ-specific expression profiling analysis, roots, mature leaves, immature leaves and stems were collected from V1 stage seedlings, and flowers, young pods (0.5 to 2 cm in length), and seeds at 14 and 21 days after flowering were collected from R3 to R6 growth stages. The same methods were followed for drought, dehydration, salt (250 mM) and ABA (150 μM) treatments [48]. IAA treatments were conducted using the same method as ABA treatments except 50 μM IAA was used instead of ABA. Shoots and roots were collected separately and tissues from three plants were pooled as one sample after drought treatments. Whole plants were collected at different time points after dehydration and salt treatments. For hormone treatments, shoots and roots from single plants were collected separately at 0.5 hour (h), 1 h, 3 h and 5 h after treatments. Samples were frozen immediately in liquid nitrogen after collection and kept at −80 °C until use.

### Quantitative RT-PCR

Total RNA extraction, design of *GmPIN* gene-specific primers for quantitative PCR (Additional file 4: Table S3), and qRT-PCR analysis were conducted following the standard methods [48]. All qPCR analyses have three biological replicates and two technical replicates.

### Promoter cloning and vector construction

Promoters (1,938 to 3,439 bp upstream of the start codon) of 10 *GmPIN*s were amplified by PCR using Phusion high-fidelity DNA polymerase (Thermo Scientific, USA), gene-specific primers (Additional file 5: Table S4), and soybean genomic DNA extracted from two-week old seedlings using the CTAB method [49]. PCR products were cloned into the Gateway pDONR™/Zeo vector (Invitrogen, USA), sequenced and then recombined into the destination vector pMDC163 [50] to produce promoter*::GUS* expression cassettes via LR reactions (Invitrogen, USA). The *DR5* synthetic promoter [51] was also constructed into pMDC163. All plant expression vectors were transformed into *Agrobacterium rhizogenes* K599 by electroporation.

### Soybean hairy root transformation and GUS staining

Soybean hairy root transformation was performed according to references with some modifications [52, 53]. Half of the Murashige & Skoog (MS) medium, with the addition of hygromycin (25 mg/L) as a selective agent for transgenic roots, was used in the hairy root subculture. For PEG and salt treatments, 250 g/L PEG 8,000 and 75 mM NaCl was added in the media. For treatments with auxin and ABA, the concentration of IAA and ABA was 10^−7^ M. Lateral roots of similar size from the same transgenic root were used for all the treatments and control. The treatments lasted for 24 hours. At least 5 independent transgenic roots were used as biological replicates.

GUS staining was performed according to the standard protocols [54]. The staining time was 30 minutes and 4 hours for *DR5::GUS* and *GmPIN* promoter*::GUS* transgenic roots, respectively. Root images were developed using a Leica S6 D stereomicroscope (Leica Microsystems, Switzerland) and Leica EC3 digital camera (Leica Microsystems, Switzerland).

## Additional files

Additional file 1: Table S1.
*GmPIN*s gene information. (PDF 81 kb) Additional file 2: Figure S1. Multiple sequence alignment of the soybean PIN gene family. The alignment was performed using the online tool-the BAR ClustalW with MView Output [55]. (PDF 8637 kb) Additional file 3: Table S2. Protein sequences used in the phylogenetic analysis. (PDF 82 kb) Additional file 4: Table S3. Primers used for the qRT-PCR analysis. (PDF 476 kb) Additional file 5: Table S4. Primers used for *GmPIN*s gene promoter cloning. (PDF 60 kb)
