# Supplementary material for: Genome-wide analysis and expression profiling of the PIN auxin transporter gene family in soybean (Glycine max)
Source: BMC Genomics. 2015 Nov 16;16:951. doi: 10.1186/s12864-015-2149-1 (PMC4647520; doi:10.1186/s12864-015-2149-1)
Supplement: Additional file 1: Table S1. — GmPINs gene information. (PDF 81 kb) [file 12864_2015_2149_MOESM1_ESM.pdf]

Table S1 *GmPIN*s gene information

Gene name	Locus ID	Protein			Chromosome	Location (Mb)	Strand direction	Transmembrane helices	Subcellular localization
		Length	MW (kDa)	PI					
GmPIN1a	Glyma08g05900	603	66.039	8.52	Gm08	4.21	+	9	plas/chlo
GmPIN1b	Glyma07g11550	605	66.192	8.52	Gm07	9.74	+	9	plas
GmPIN1c	Glyma09g30700	605	66.307	8.33	Gm09	37.48	-	9	plas
GmPIN1d	Glyma03g28130	597	64.260	9.04	Gm03	36.01	+	8	plas
GmPIN1e	Glyma19g30900	578	62.023	8.55	Gm19	38.59	+	8	plas
GmPIN2a	Glyma13g00390	642	69.746	9.07	Gm13	0.14	+	9	plas
GmPIN2b	Glyma17g06460	637	69.033	9	Gm17	4.61	+	9	plas
GmPIN3a	Glyma07g34190	665	72.117	7.84	Gm07	39.10	-	9	plas
GmPIN3b	Glyma20g01760	666	75.517	7.54	Gm20	1.29	-	10	plas
GmPIN3c	Glyma07g22340	608	66.335	7.5	Gm07	23.72	+	9	plas
GmPIN3d	Glyma09g20580	634	69.338	7.59	Gm09	25.49	+	10	plas
GmPIN5a	Glyma09g38700	377	41.460	7.59	Gm09	44.00	-	9	vacu
GmPIN5b	Glyma18g47630	369	40.656	8.02	Gm18	57.23	+	9	vacu
GmPIN6a	Glyma13g09030	478	52.123	7.29	Gm13	10.03		9	plas/vacu
GmPIN6b	Glyma14g27900	531	85.120	8.62	Gm14	34.23	+	9	plas
GmPIN8a	Glyma05g23180	362	40.053	8.87	Gm05	28.77	+	8	chlo
GmPIN8b	Glyma17g16870	363	40.192	8.87	Gm17	13.67	-	8	chlo
GmPIN8c	Glyma09g37561	353	38.694	9.83	Gm09	43.09	+	8	chlo
GmPIN8d	Glyma18g49080	359	39.486	9.74	Gm18	58.47	-	8	plas
GmPIN9a	Glyma09g06970	443	49.504	9.07	Gm09	5.80	-	8	plas
GmPIN9b	Glyma15g18241	469	51.749	9.18	Gm15	14.90	-	5	cyto
GmPIN9c	Glyma09g13500	460	81.070	9.14	Gm09	14.98	+	9	plas
GmPIN9d	Glyma15g25690	492	54.290	8.02	Gm15	27.23	+	9	plas
