# Supplementary material for: Genome-wide analysis and expression profiling of the PIN auxin transporter gene family in soybean (Glycine max)
Source: BMC Genomics. 2015 Nov 16;16:951. doi: 10.1186/s12864-015-2149-1 (PMC4647520; doi:10.1186/s12864-015-2149-1)

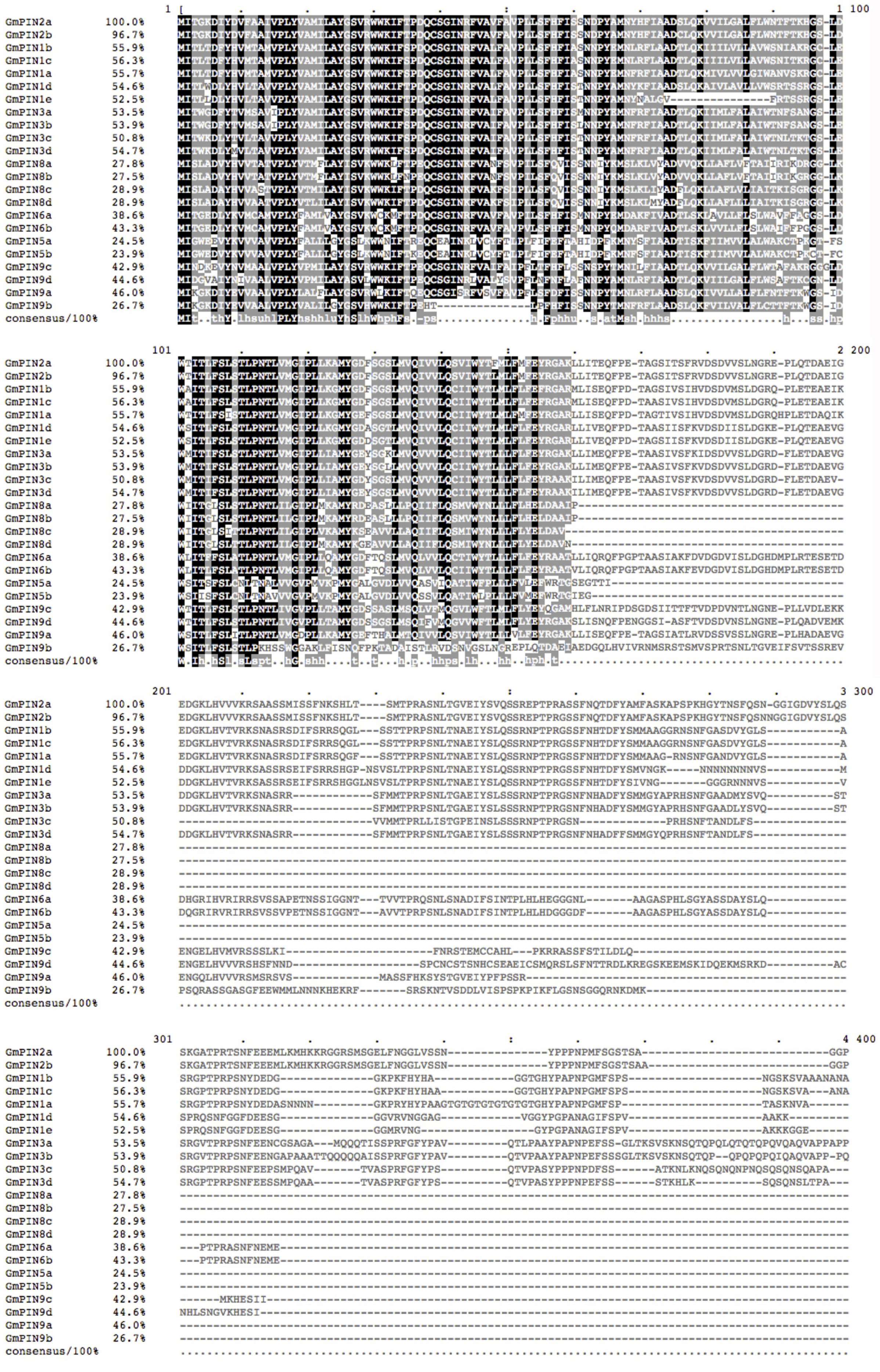

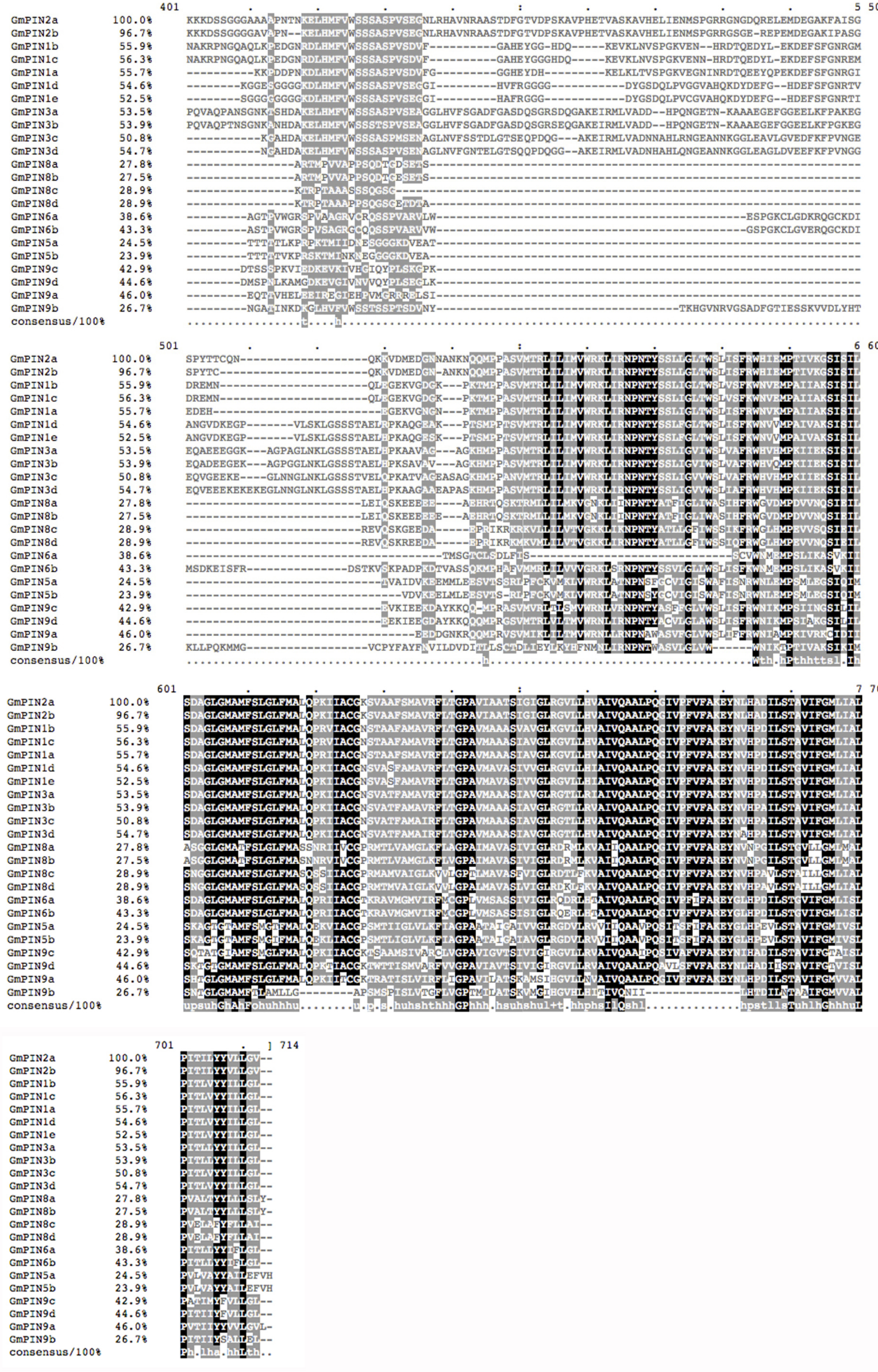

Supplement: Additional file 2: Figure S1. — Multiple sequence alignment of the soybean PIN gene family. The alignment was performed using the online tool-the BAR ClustalW with MView Output [55]. (PDF 8637 kb) [file 12864_2015_2149_MOESM2_ESM.pdf]
