# Supplementary material for: Genome-wide analysis and expression profiling of the PIN auxin transporter gene family in soybean (Glycine max)
Source: BMC Genomics. 2015 Nov 16;16:951. doi: 10.1186/s12864-015-2149-1 (PMC4647520; doi:10.1186/s12864-015-2149-1)
Supplement: Additional file 3: Table S2. — Protein sequences used in the phylogenetic analysis. (PDF 82 kb) [file 12864_2015_2149_MOESM3_ESM.pdf]

Table S2 Protein sequences used in the phylogenetic analysis

Species	PINs	ID number[a]	Other name [16, 20]
Glycine max			
	GmPIN1a	Glyma08g05900.1	
	GmPIN1b	Glyma07g11550.1	
	GmPIN1c	Glyma09g30700.1	
	GmPIN1d	Glyma03g28130.2	
	GmPIN1e	Glyma19g30900.2	
	GmPIN2a	Glyma13g00390.1	
	GmPIN2b	Glyma17g06460.1	
	GmPIN3a	Glyma07g34190.2	
	GmPIN3b	Glyma20g01760.1	
	GmPIN3c	Glyma07g22340.2	
	GmPIN3d	Glyma09g20580.1	
	GmPIN5a	Glyma09g38700.2	
	GmPIN5b	Glyma18g47630.1	
	GmPIN6a	Glyma13g09030.1	
	GmPIN6b	Glyma14g27900.1	
	GmPIN8a	Glyma05g23180.1	
	GmPIN8b	Glyma17g16870.2	
	GmPIN8c	Glyma09g37561.1	
	GmPIN8d	Glyma18g49080.1	
	GmPIN9a	Glyma09g06970.2	
	GmPIN9b	Glyma15g18241.1	
	GmPIN9c	Glyma09g13500.1	
	GmPIN9d	Glyma15g25690.1	
Phaseolus vulgaris			
	PvPIN1a	Phvul.002G330300.1	
	PvPIN1b	Phvul.004G150600.1	
	PvPIN1c	Phvul.011G164500.1	
	PvPIN1d	Phvul.001G121100.1	
	PvPIN2	Phvul.003G139600.1	
	PvPIN3a	Phvul.002G094000.1	
	PvPIN3b	Phvul.003G032500.1	
	PvPIN5	Phvul.008G055200.1	
	PvPIN6	Phvul.001G063900.1	
	PvPIN8a	Phvul.004G026500.1	
	PvPIN8b	Phvul.008G043700.1	
	PvPIN9a	Phvul.009G210900.1	
	PvPIN9b	Phvul.009G234000.1	
	PvPIN9c	Phvul.009G234100.1	

	PvPIN9d	Phvul.009G234200.1	
	PvPIN9e	Phvul.009G208200.1	
Medicago truncatula			
	MtPIN1a	Medtr6g069510.1	MtPIN4
	MtPIN1b	Medtr8g107360.1	MtPIN5
	MtPIN1c	Medtr7g089360.1	
	MtPIN2a	Medtr4g127100.1	MtPIN2
	MtPIN2b	Medtr4g127090.1	MtPIN7
	MtPIN3a	Medtr4g084870.1	MtPIN1
	MtPIN3b	Medtr1g030890.1	MtPIN3
	MtPIN5a	Medtr7g079720.1	
	MtPIN5b	Medtr2g038470.1	
	MtPIN6	Medtr1g029190.1	MtPIN6
	MtPIN8a	Medtr6g011400.1	
	MtPIN8b	Medtr7g009370.1	MtPIN8
Lotus japonicus			
	LjPIN1a	chr4.CM0042.2390.r2.m	
	LjPIN1b	chr1.CM0269.740.r2.m	
	LjPIN2	chr4.CM0244.800.r2.m	
	LjPIN5	chr1.CM1413.300.r2.m	
	LjPIN8	chr2.CM0081.1950.r2.m	
Arabidopsis thaliana			
	AtPIN1	AT1G73590.1	
	AtPIN2	AT5G57090.1	
	AtPIN3	AT1G70940.1	
	AtPIN4	AT2G01420.2	
	AtPIN5	AT5G16530.1	
	AtPIN6	AT1G77110.1	
	AtPIN7	AT1G23080.1	
	AtPIN8	AT5G15100.1	
Oryza sativa			
	OsPIN1a	LOC_Os06g12610.1	
	OsPIN1b	LOC_Os02g50960.1	
	OsPIN1c	LOC_Os11g04190.1	
	OsPIN1d	LOC_Os12g04000.1	
	OsPIN2	LOC_Os06g44970.1	
	OsPIN5a	LOC_Os01g69070.1	
	OsPIN5b	LOC_Os08g41720.1	
	OsPIN5c	LOC_Os09g32770.1	
	OsPIN8	LOC_Os01g51780.1	

	OsPIN9	LOC_Os01g58860.1	
	OsPIN10a	LOC_Os01g45550.1	
	OsPIN10b	LOC_Os05g50140.1	
Zea mays			
	ZmPIN1a	GRMZM2G098643	
	ZmPIN1b	GRMZM2G074267	
	ZmPIN1c	GRMZM2G149184	
	ZmPIN1d	GRMZM2G171702	
	ZmPIN2	AZM5_105354 & AZM5_3988	
	ZmPIN5a	GRMZM2G025742	
	ZmPIN5b	GRMZM2G148648	
	ZmPIN5c	GRMZM2G040911	
	ZmPIN8	GRMZM5G839411	
	ZmPIN9	GRMZM5G859099	
	ZmPIN10a	GRMZM2G126260	
	ZmPIN10b	GRMZM2G160496	
Sorghum bicolor			
	SbPIN1a	Sb10g008290.1	SbPIN10
	SbPIN1b	Sb04g028170.1	SbPIN6
	SbPIN1c	Sb05g002150.1	SbPIN7
	SbPIN2	Sb10g026300.1	SbPIN11
	SbPIN5a	Sb03g043960.1	SbPIN5
	SbPIN5b	Sb07g026370.1	SbPIN8
	SbPIN5c	Sb02g029210.1	SbPIN1
	SbPIN8	Sb03g032850.1	SbPIN3
	SbPIN9	Sb03g037350.1	SbPIN4
	SbPIN10a	Sb03g029320.1	SbPIN2
	SbPIN10b	Sb10g004430.1	SbPIN9

[a]Protein sequences from all plant species were retrieved from phytozome (V9.1) except *Lotus japonicus* (http://www.kazusa.or.jp/lotus/).
