# Supplementary material for: Genome-wide analysis and expression profiling of the PIN auxin transporter gene family in soybean (Glycine max)
Source: BMC Genomics. 2015 Nov 16;16:951. doi: 10.1186/s12864-015-2149-1 (PMC4647520; doi:10.1186/s12864-015-2149-1)
Supplement: Additional file 4: Table S3. — Primers used for the qRT-PCR analysis. (PDF 476 kb) [file 12864_2015_2149_MOESM4_ESM.pdf]

Table S3 Primers used for qRT-PCR analysis

Gene name	Locus ID	Forward primer (5'-3')	Reverse primer (5'-3')
GmPIN1a	Glyma08g05900	GCAACCGAGGATCATAGCAT	AGAGAACGCCTTTGAGTCCA
GmPIN1b	Glyma07g11550	TGTTGATTGCTTTGCCCATA	TGTCTGATGCTCAACAAGCC
GmPIN1c	Glyma09g30700	ATGCAAAGCTTGGTTGAGGT	GATCCTGGGGTTCTTCTTCC
GmPIN1d	Glyma03g28130	GCATAAAAAGTGGGACCGAA	ATGACAACCTGTGCCATTCA
GmPIN1e	Glyma19g30900	GGGATGCTAATTGCTCTTCCTA	GGTAGTTTGATCCACACTGCAA
GmPIN2a	Glyma13g00390	ATTGCATTGCCCATAACCAT	CGTGACCTTGGGTTTACGTT
GmPIN2b	Glyma17g06460	ATTGCATTGCCCATAACCAT	CGTGACATTGGGTTTACATAG
GmPIN3a	Glyma07g34190	AATAAAACCAGCCACGATGC	ATCCGATGCTCCAAAATCAG
GmPIN3b	Glyma20g01760	AGAATTCGCAGACACAGCCT	GTTGGCTTTGTTCCCACTGT
GmPIN3c	Glyma07g22340	GTGACGGTAGCTTCTCCTCG	GAATTCTGGCTCTGGCTCTG
GmPIN3d	Glyma09g20580	ACACTTGCAAAATGGGGAAG	GCCCAACTTGTTGAGTCCAT
GmPIN5a	Glyma09g38700	ATCGTTGTAGGATTGCGAGG	TCATACCAAAGATCACCGCA
GmPIN5b	Glyma18g47630	GGTGCCATTGCTGTAGGATT	TCATACCAAAGATCACCGCA
GmPIN6a	Glyma13g09030	TGCAACTCGTGGTTCTTCAG	CGTCGAATTTCGCTATGGAT
GmPIN6b	Glyma14g27900	AGTTCTTGACATGCCCTGCT	TTCCCACAAGCTTTTCCAAC
GmPIN8a	Glyma05g23180	AGCCGATGATCCAAGAGAGA	ACAGCCATCCAATTCAAACA
GmPIN8b	Glyma17g16870	TGTCCGAAGTGGACTGTTGA	CAACCAACCTCCAGCAAACT
GmPIN9d	Glyma15g25690	GCCATCAATTGCAAAAGGTT	GAACCGAGCCACCATAGAAA
GmUBI3	Glyma20g27950	GTGTAATGTTGGATGTGTTCCC	ACACAATTGAGTTCAACACAAACCG
