# Supplementary material for: Genome-wide analysis and expression profiling of the PIN auxin transporter gene family in soybean (Glycine max)
Source: BMC Genomics. 2015 Nov 16;16:951. doi: 10.1186/s12864-015-2149-1 (PMC4647520; doi:10.1186/s12864-015-2149-1)
Supplement: Additional file 5: Table S4. — Primers used for GmPINs gene promoter cloning. (PDF 60 kb) [file 12864_2015_2149_MOESM5_ESM.pdf]

Table S4 Primers used for *GmPIN*s gene promoter cloning

Gene name	Locus ID	Forward primer (5'-3')	Reverse primer (5'-3')	Fragment length (bp)
GmPIN1b	Glyma07g11550	ATTTGTGGGTTTGTGAGGT	TGTAATTGAAGAATGGTGTAAAAG	2511
GmPIN1c	Glyma09g30700	AGAAAGATGATAGAAGCAGAAGAG	GAGAGAACAAAAAAATGTTAAAGGA	2763
GmPIN1e	Glyma19g30900	GCTTCCATCAAACCCTTTG	TGTCGTGTCTCTTTCCGAATA	2887
GmPIN2a	Glyma13g00390	GTTCATGCGGGGTTTGT	CGATTGAGGAAGTAGTCCTTT	1938
GmPIN2b	Glyma17g06460	AAGGATTGGGTTTATGGATTAG	GGTTAATGCAGGGTTTGTAG	2721
GmPIN3a	Glyma07g34190	AAGGACCCATGAAACAATAATT	TTTGCCTTTTTCTTAATTTCTAAA	3439
GmPIN3b	Glyma20g01760	TTCCCTAAGATTCCTCAAGCTT	TTTCCCCTTTCTCTTAATTTCTC	3368
GmPIN6a	Glyma13g09030	GAGAGAAAATTACTAAGTGTGTTTGG	AGAAGGAATAAAATTGGAAAATAATAA	2969
GmPIN6b	Glyma14g27900	GAAACATCACAACAGAGACAGA	CTCGAGAGAGAATTTTTATAGTGTATTT	3486
GmPIN9d	Glyma15g25690	GTGACAGCGAGAGATCGAA	GAAGATGATAGGTACAAGTTAAAGA	2132
